# Severe Cord Entanglement in a Monochorionic–Monoamniotic Twin Pregnancy: Highlighting Perinatal Risks and Management Challenges—A Case Report

**DOI:** 10.1155/crog/5549397

**Published:** 2026-03-08

**Authors:** Michel Alagha, Karmen Saroufine, Hassan Aji, Ghassan Nabbout

**Affiliations:** ^1^ Department of Biomedical Sciences, Faculty of Medicine and Medical Sciences, University of Balamand, Al-Koura, Lebanon, balamand.edu.lb; ^2^ Department of Obstetrics and Gynecology, Nini Hospital, Tripoli, Lebanon, hopitalnini.com

**Keywords:** entanglement, monochorionic–monoamniotic pregnancies, prenatal diagnosis, twin pregnancy complications, umbilical cord

## Abstract

Monochorionic–monoamniotic (MCMA) twin pregnancies represent the rarest and highest risk form of twin gestation, largely due to complications such as umbilical cord entanglement (UCE). We report a case of a 27‐year‐old woman with a spontaneously conceived MCMA twin pregnancy complicated by an extreme intraoperative finding of 17 loops of UCE, yet with a favorable neonatal outcome. MCMA gestation was diagnosed antenatally by ultrasound demonstrating a single placenta without an intertwin membrane. The patient was admitted at 31 weeks′ gestation for planned inpatient monitoring and administration of antenatal corticosteroids, followed by elective cesarean delivery at 32 weeks′ gestation. Both female neonates were delivered in vertex presentation and admitted to the neonatal intensive care unit (NICU) due to prematurity. Birthweights, Apgar scores, and short‐term neonatal outcomes were reassuring, with no major complications noted prior to discharge. This case underscores the importance of early diagnosis, close fetal surveillance, and appropriately timed delivery in MCMA pregnancies. Despite the high risk associated with UCE, favorable outcomes can be achieved through vigilant prenatal care and multidisciplinary management.

## 1. Introduction

Monochorionic–monoamniotic (MCMA) twin pregnancies represent the rarest and most hazardous form of twin gestation. They result from monozygotic division occurring between days 8 and 12 after fertilization, leading to a shared placenta and a single amniotic sac. Although assisted reproductive technologies have been associated with an increased incidence of monozygotic twinning, most MCMA pregnancies remain spontaneously conceived [1].

MCMA twins account for approximately 1% of all twin pregnancies and 5% of monochorionic gestations, with an estimated incidence of 1 in 10,000 pregnancies [2]. The absence of an intertwin membrane exposes the fetuses to unique risks, including preterm birth, twin‐to‐twin transfusion syndrome, fetal growth restriction, and particularly umbilical cord complications. Among these, umbilical cord entanglement (UCE) is almost universal in MCMA pregnancies and represents the leading cause of sudden intrauterine fetal demise [3].

Advances in ultrasonography and Doppler imaging have improved early recognition of MCMA pregnancies and allowed antenatal visualization of cord entanglement, sometimes as early as the first trimester [4]. Nevertheless, prenatal identification of UCE does not reliably predict adverse outcomes, as entanglement may remain clinically silent or acutely worsen without warning. Consequently, management strategies emphasize intensive fetal surveillance and planned preterm delivery to mitigate the risk of catastrophic cord accidents.

We present a rare case of a spontaneously conceived MCMA twin pregnancy with an exceptionally severe intraoperative finding of a 17‐loop UCE, yet with a favorable neonatal outcome following elective cesarean delivery at 32 weeks′ gestation.

## 2. Case Presentation

A 27‐year‐old woman, gravida 5 para 3 abortus 1 (G5P3A1), blood group O positive, presented for her first prenatal visit after a positive home pregnancy test. Her last menstrual period was uncertain, and conception was spontaneous. She had attended nine routine antenatal visits at our hospital. During early pregnancy, she experienced hyperemesis gravidarum, which resolved with conservative management. No chronic medical conditions or obstetric complications were reported thereafter. Due to a previous miscarriage, she was prescribed low‐dose aspirin throughout pregnancy. Ultrasound examinations at 13 and 22 weeks′ gestation demonstrated a twin pregnancy with a single anterior–fundal placenta and absence of an intertwin dividing membrane, confirming an MCMA configuration. Both fetuses were viable, structurally normal, and in cephalic presentation. Estimated fetal weights were appropriate for gestational age, and umbilical artery Doppler studies were normal. No antenatal sonographic evidence of cord entanglement was identified.

At 31 weeks′ gestation, the patient was electively admitted for inpatient monitoring due to the known high‐risk nature of MCMA pregnancy. On admission, maternal vital signs were stable (blood pressure 110/70 mmHg, heart rate 82 bpm, afebrile), and no uterine contractions or vaginal bleeding was present. Fetal surveillance consisted of daily cardiotocography and serial ultrasound assessments, all of which demonstrated reassuring fetal heart rate patterns.

A course of antenatal corticosteroids was administered (four intramuscular doses of dexamethasone 6 mg at 12‐h intervals over 48 h) to promote fetal lung maturation. Following multidisciplinary counseling and in accordance with institutional protocol for MCMA pregnancies, elective cesarean delivery was scheduled at 32 weeks′ gestation to reduce the risk of sudden intrauterine fetal demise.

**FIGURE 1:**
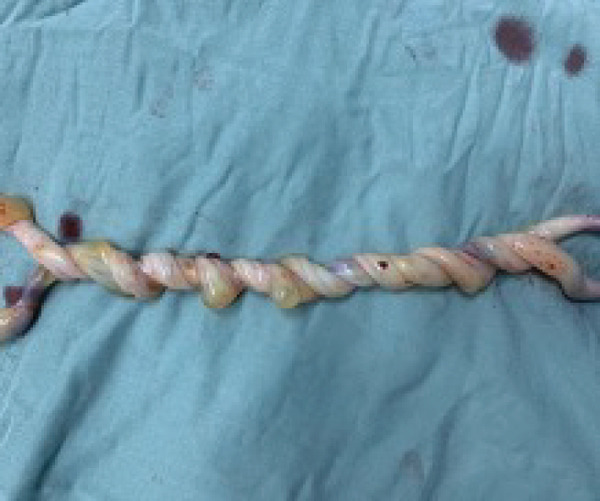
Twisting of umbilical cords.

**FIGURE 2:**
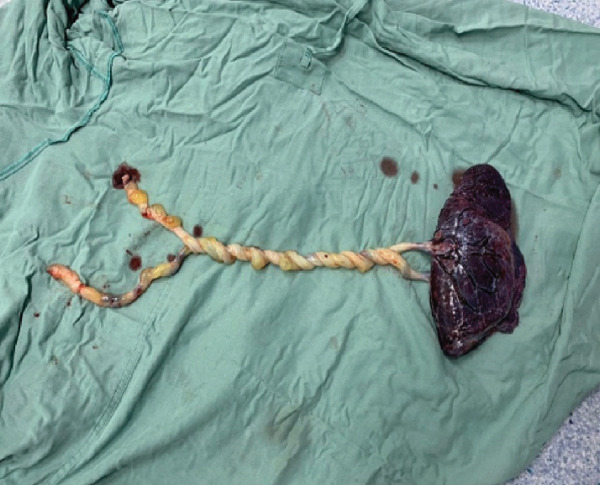
Twisting of umbilical cords from a shared placenta.

During the procedure, two live female neonates were delivered, both in vertex presentation. Intraoperative inspection revealed a striking finding of severe UCE consisting of 17 distinct loops, involving both cords tightly coiled around each other (Figures 1 and 2). The number of loops was determined by direct visual counting after complete delivery of the placenta and cords. No true knots or macroscopic evidence of thrombosis or vascular compromise were observed. Cord length was not formally measured.

Twin A weighed 1720 g, and Twin B weighed 1680 g. Apgar scores were 8 and 9 at 1 and 5 min, respectively, for both neonates. Neither required intubation; both received short‐term continuous positive airway pressure (CPAP) support for prematurity‐related respiratory adaptation. Cranial ultrasonography performed during NICU stay showed no intraventricular hemorrhage or structural abnormalities.

Both neonates remained in the NICU for 18 days, primarily for feeding maturation and weight gain, and were discharged in stable condition. Short‐term follow‐up at discharge revealed normal neurological examination; longer term neurodevelopmental follow‐up was not yet available at the time of reporting.

### 2.1. Timeline of Clinical Events

• 13 weeks′ gestation: First‐trimester ultrasound showing twin pregnancy with a single placenta and no dividing membrane
• 22 weeks′ gestation: Repeat ultrasound confirming MCMA configuration; normal growth and amniotic fluid
• 31 weeks′ gestation: Hospital admission for planned inpatient surveillance; maternal vitals stable; antenatal corticosteroids administered
• 31 weeks + 2 days: Completion of dexamethasone course
• 31–32 weeks′ gestation: Reassuring cardiotocography and ultrasound monitoring
• 32 weeks′ gestation: Elective cesarean section due to MCMA pregnancy and risk of acute cord accident
• Postnatal period: NICU admission for prematurity; uncomplicated course; discharge in good condition

## 3. Discussion

MCMA twin pregnancies carry the highest perinatal risk among twin gestations, with reported mortality rates ranging from 12% to 23% despite modern surveillance strategies [5]. This is largely due to complications such as preterm birth, twin‐to‐twin transfusion syndrome (TTTS), intrauterine growth restriction (IUGR), and, most notably, umbilical cord complications, including knots, compression, and entanglement [4].

UCE is nearly universal in MCMA pregnancies and remains the principal mechanism underlying sudden fetal compromise and intrauterine death [6].

Cord entanglement may result in intermittent or acute occlusion of umbilical blood flow, leading to hypoxia, acidosis, and neurological injury. Although protective features such as Wharton′s jelly and the elasticity of umbilical vessels may initially preserve perfusion, progressive tightening or acute traction can precipitate catastrophic fetal events [7]. Importantly, the severity of entanglement observed at delivery does not always correlate with antenatal findings or clinical deterioration.

In the present case, no antenatal ultrasonographic evidence of cord entanglement or Doppler abnormalities was identified, highlighting the diagnostic limitations of imaging and the unpredictable nature of cord accidents in MCMA pregnancies. Similar observations have been reported in both high‐resource and low‐resource settings. Recent evidence from resource‐limited settings shows that positive outcomes in MCMA pregnancies complicated by cord issues are possible when diagnosis is timely and appropriate interventions are carried out. For instance, Minayehu et al. [8] reported a case from a low‐ and middle‐income country where prenatal ultrasound identified cord compression in an MCMA twin pregnancy [8]. This led to closer monitoring and a well‐timed delivery, resulting in healthy neonates. Their experience demonstrates that, even in environments with limited resources, organized antenatal care and prompt obstetric decisions can markedly lower perinatal complications and mortality.

In contrast, our case did not reveal clear sonographic evidence of cord entanglement before delivery, highlighting how prenatal detection of such complications can be inconsistent. This reinforces that a lack of imaging findings does not eliminate the risk. Collectively, these observations support current guidelines that recommend vigilant inpatient monitoring and planned early delivery in MCMA pregnancies to reduce the unpredictable danger of acute cord accidents, regardless of the healthcare setting [8].

Given the high risk of sudden fetal demise, most contemporary guidelines recommend planned inpatient monitoring starting between 26 and 28 weeks′ gestation and elective cesarean delivery between 32 and 34 weeks, balancing the risks of prematurity against the risk of intrauterine death [9–12]. In our case, elective delivery at 32 weeks followed institutional protocol and was supported by reassuring fetal surveillance and completion of antenatal corticosteroids.

The exceptional finding of 17 loops of UCE underscores the remarkable adaptability of the fetoplacental circulation and reinforces that favorable outcomes are achievable even in extreme anatomical scenarios when appropriate surveillance and timing of delivery are applied.

## 4. Conclusion

This case highlights the severe yet unpredictable nature of UCE in MCMA twin pregnancies. Despite an extreme 17‐loop cord entanglement, favorable neonatal outcomes were achieved through early diagnosis, intensive inpatient surveillance, and timely elective cesarean delivery. Adherence to evidence‐based management protocols and multidisciplinary care remains essential to optimizing outcomes in this highest risk form of twin gestation.

## Author Contributions

**Michel Alagha:** data curation, writing – original draft preparation. **Karmen Saroufine:** data curation, writing – original draft preparation. **Hassan Aji:** conceptualization, supervision. **Ghassan Nabbout:** supervision, writing – review and editing.

## Funding

No funding was received for this manuscript.

## Ethics Statement

This study was approved by the ethics committee of Nini Hospital. Written informed consent was obtained from the patient.

## Conflicts of Interest

The authors declare no conflicts of interest.

## Patient Perspective

The decision for a cesarean section at 32 weeks′ gestation was stressful enough without being aware of the cord entanglement. Couldn′t believe I was at risk of losing my babies especially after a previous miscarriage. I cannot thank the doctors enough for their compassion and professionalism.

## Data Availability

The data that support the findings of this study are available from the corresponding author upon reasonable request.
